# Immunopathological insights into endometriosis: from research advances to future treatments

**DOI:** 10.1007/s00281-025-01058-5

**Published:** 2025-07-18

**Authors:** Yangyang Dai, Zi Ye, Xiang Lin, Songying Zhang

**Affiliations:** 1 Assisted Reproduction Unit, Department of Obstetrics and Gynecology, Sir Run Run Shaw Hospital, School of Medicine, Zhejiang University, Hangzhou, 310016 China; 2 Zhejiang Provincial Clinical Research Center for Reproductive Health and Disease, Hangzhou, 310016 China; 3 Zhejiang Key Laboratory of Precise Protection and Promotion of Fertility, Hangzhou, 310016 China

**Keywords:** Endometriosis, Immune, Inflammation, Fertility, Immunotherapy

## Abstract

Endometriosis is a chronic gynecological disease and a major global concern for women’s health. With advancing knowledge of the condition, the classic definition of endometriosis as “endometrium-like tissue outside the uterus” now appears insufficient to explain its pathophysiology, as it overlooks the complex involvement of multiple systems in disease development. Immunological changes have been recognized in endometriosis for decades, and growing evidence substantiates that immunopathological alterations are a hallmark of the disease. Imbalanced immune cell populations and cellular dysfunctions within both the innate and adaptive immune systems, along with aberrant inflammatory cytokines, contribute to the inflammation associated with endometriosis. Moreover, immune cell dysfunctions such as reduced natural killer (NK) cell activity, impaired dendritic cell (DC) maturation and inhibited T cell function via immune checkpoints (ICPs) make the microenvironment also immune-suppressive, facilitating the immune evasion of endometriotic lesions. Endometriosis associated inflammation also sabotages female fertility across multiple stages, including ovarian function, fertilization, embryo development and pregnancy complications. Recognition of the inflammatory and immune-suppressive microenvironment associated with endometriosis leads to the discovery of potential immunotherapeutic targets. Established treatments like non-steroid anti-inflammatory drugs (NSAIDs) and hormone therapies harbor immunomodulatory properties. Other immune-based therapies such as immune cell therapies, cytokine-targeting therapies and immune checkpoint inhibitors (ICIs), which have demonstrated significant efficacy in many chronic inflammatory diseases including cancers, may hold substantial promise as future treatments for endometriosis.

## Introduction

Endometriosis is a chronic gynecological disease affecting millions of women of reproductive age and causing huge financial burden worldwide. The pathophysiology of endometriosis is complicated and remains elusive. Sampson’s theory, the most widely accepted theory of endometriosis pathogenesis, proposes that retrograde menstruation through fallopian tubes to the peritoneal cavity leads to the development of endometriosis. However, endometriosis is not limited to the pelvis and has systemic effects. Immunopathological changes are prominent in endometriosis, with immune cell dysfunction and abnormal inflammatory mediators contributing to both local inflammation (lesions, peritoneal cavity, etc.) and systemic inflammation. Endometriosis is now also recognized as a chronic estrogen-dependent inflammatory disease. Whether inflammation instigates and/or perpetuates endometriosis is still unknown, but increasing evidence indicates that targeting inflammation may represent a novel therapeutic strategy for endometriosis [1, 2].

The immune microenvironment of endometriosis is complicated and dynamic during disease progression. Cells in both the innate immune system and adaptive immune system are present in endometriotic lesions and contribute to the immunopathological changes. Immune cell abnormalities associated with endometriosis include accumulation of disease-associated macrophages (DAMs), reduced natural killer (NK) cell activity, hampered dendritic cell maturation, and inhibited T cell cytotoxicity etc. Aberrant cytokines are concurrent with cell dysfunction.

**Fig. 1:**
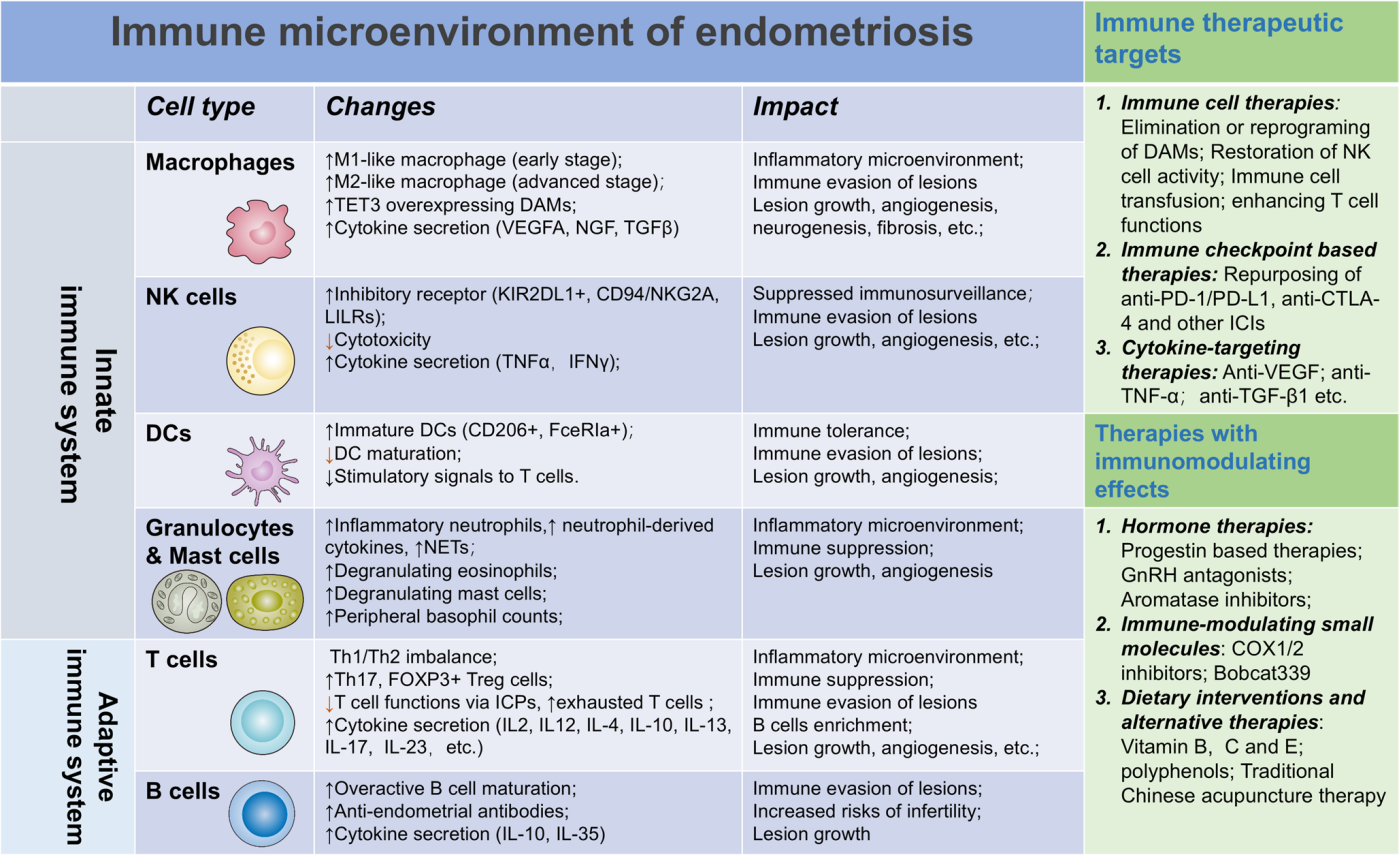
Summary of the immune microenvironment of endometriosis, and possible immune therapeutic targets

**Fig. 2:**
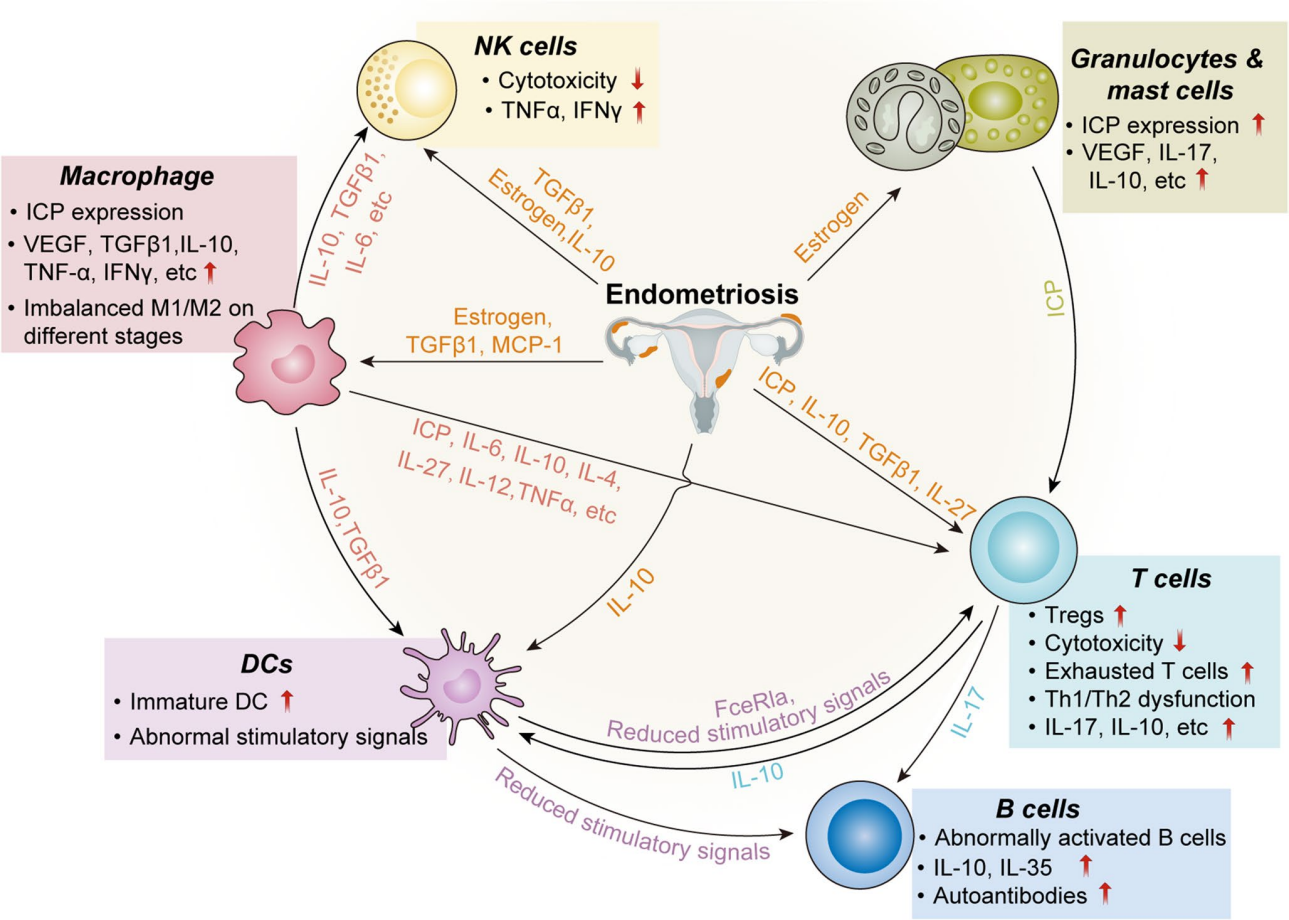
Interrelationship of immune cells in endometriosis: disease associated changes accompanied with endometriosis influence immune cell behaviors, altering their phenotypes and functions. The aberrant cytokine production and cell surface ligand/receptors (e.g. ICPs) expression on various immune cells form an intricate modulatory network which furtherly exacerbate the inflammatory and immune suppressive microenvironment of endometriosis

**Fig. 3:**
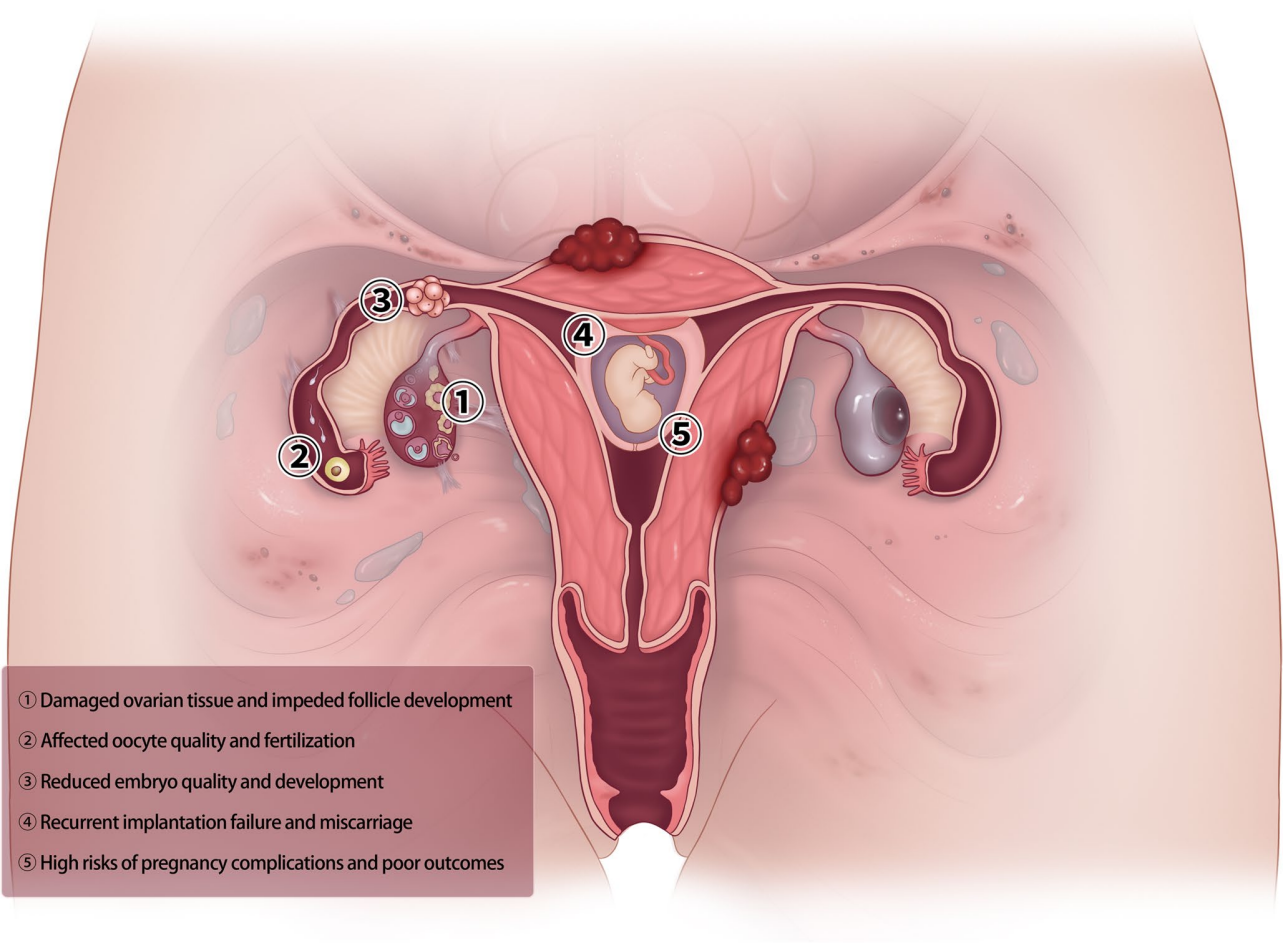
Endometriosis-associated inflammation impairs female fertility. ① Damaged ovarian tissue and impeded follicle development,② Affected oocyte quality and fertilization, ③ Reduced embryo quality and development, ④ Recurrent implantation failure and miscarriage, ⑤ High risks of pregnancy complications and poor outcomes

In this article, we review the immunopathological changes in endometriosis by the cell types in the innate and adaptive immune systems (Figs. 1 and 2) and summarize the possible mechanisms by which endometriosis-associated inflammation may impair female fertility (Fig. 3), and discuss possible therapeutic targets (Fig. 1).

## Innate immune cells in endometriosis

### Macrophages

The disease microenvironment can recruit and educate monocytes/macrophages into DAMs. The pro-disease and anti-disease functions of DAMs in tumors, auto-immune diseases, tissue regeneration, and endometriosis have been widely investigated.

Macrophages in endometriotic lesions have multiple origins. In Sampson’s retrograde menstruation theory, the refluxed endometrium tissue contains cells from the eutopic endometrium; thus, macrophages derived from the eutopic endometrium would be expected in the endometriotic lesion. However, defining these macrophages in human samples is difficult. Linear tracing studies in mouse models proved that both eutopic endometrial macrophages and infiltrating macrophages contributed to the macrophage pool in peritoneal endometriotic lesions, and infiltrating macrophages could be of multiple origins such as peritoneal macrophages and/or blood infiltrated macrophages [3]. Early clinical studies provided evidence for the participation of peripheral monocytes in endometriosis. Akoum et al. observed that the concentration and chemotactic activity of monocyte chemotactic protein-1 (MCP-1) were elevated in the peripheral blood and endometrium of endometriosis patients, which suggested active monocyte chemotaxis during endometriosis [4]. More direct evidence was reported in animal studies, which showed that bone marrow-derived macrophages accumulated in implanted endometriotic lesions and promoted lesion growth [5]. Hogg et al. demonstrated that peritoneal macrophages also infiltrate into endometriotic lesions along with eutopic endometrium-derived macrophages and monocytes. Moreover, their results suggest that macrophages of different origins have distinct effects on endometriosis development [3].Considering most of the endometriosis animal models mimic peritoneal endometriosis or pelvic endometriosis, other forms of endometriosis are likely to have nonidentical macrophage origins. However, the current studies have shown that endometriosis-associated macrophages have a complicated ontogeny and may play different roles in endometriosis development depending on their origin.

Single-cell analysis revealed that eutopic endometrium and endometriotic lesions shared some macrophage populations but differed in others, and the proportions of the macrophages were intrinsically different [6]. Blood-infiltrated, possibly monocyte-derived, macrophages were present in all samples, with the highest density in non-endometriotic eutopic endometrium. Tissue-resident macrophages were also present in all samples, with higher proportions in peritoneal and ovarian endometriotic lesions. Peritoneal macrophages (also tissue-resident) were only detected in peritoneal endometriotic lesions, suggesting their specific role in the pathogenesis of peritoneal endometriotic lesions. These results suggest that different forms of endometriosis exhibit similarities and differences in the immune microenvironment.

The previously widely accepted M1/M2 nomenclature (M1, classically activated; M2, alternatively activated) faced refutation because it oversimplified the complexity and ignored the versatility of macrophages. One advantage of the M1/M2 nomenclature is that it allows researchers to capture a quick and overall characteristic of the disease microenvironment. Previous research adopted this nomenclature system and discovered that an M1/M2 imbalance is common in endometriosis. An increased proportion of M2-like macrophages was observed in endometriotic tissues and peritoneal fluids. In the peritoneal fluid from endometriosis patients, a proportion of macrophages showed lower levels of classic macrophage markers CD14 and CD68, and these macrophages exhibited M2-like behaviors [7, 8]. Another study reported that the ratio of C163/CD86 macrophages in the peritoneal fluid of endometriotic patients was increased, and this ratio was higher in advanced stages (III–IV) compared with early stages (I–II) [9]. CD206, another M2 macrophage marker, was also reported to be increased in early stage endometriosis [10]. One report demonstrated an increased number of CD163 + CD206 + M2 macrophages in endometriotic lesions compared with eutopic endometrium [11], and these macrophages may form a positive cellular circuit with endometriotic stromal cells where they favor each other’s prosperity [12]. Single-cell analysis also supported a suppression of classically activated macrophages and an enrichment of alternatively activated macrophages in endometriotic lesions [6, 13]. The alternatively activated macrophages are believed to facilitate endometriotic lesion formation by secreting trophic factors, such as vascular endothelial growth factor (VEGF), nerve growth factor (NGF), and transforming growth factor-β1 (TGF-β1), promoting angiogenesis, neurogenesis, and fibrogenesis at the lesion sites [14]. Moreover, the predominance of alternatively activated macrophages would tilt the microenvironment to an immunosuppressive status. These results suggested that M2 macrophage dominance and endometriosis development may be a reciprocal process.

Animal experiments validated the role of M2-like macrophages in endometriosis. In mice with induced peritoneal endometriosis, CD163 + macrophages accumulated in lesions over time [15]. Adaptive transfusion of M0, M1, and M2 macrophages led to differences in lesion progression in mouse models: M0 macrophages did not affect lesion weight; M1 macrophages significantly decreased lesion weight; and M2 macrophages caused an increase in lesion weight [16]. In another CD206 diphtheria toxin receptor mouse, which enables the depletion of CD206 + cells with diphtheria toxin, Ono and colleagues observed significantly decreased endometriotic lesion sizes and endometriotic cell proliferation and angiogenesis compared with the control group; VEGFA and TGF-β1 levels were also lower in the depleted mice [17]. While most evidence from animal experiments was consistent with the major clinical observation that M2 macrophages contribute to endometriosis progression, M1 macrophages or an overlapping of M1/M2 phenotype was also noted. In the early phase (2–3 weeks) after endometriosis induction, iNOS + M1 macrophages showed an ascending trend before CD163 + macrophages began to accumulate at 4–5 weeks [15], which interestingly coincided with some clinical studies that M1 macrophage markers were enriched in the early stages of endometriosis [10, 16]. This clear overlapping expression of M1 and M2 markers may suggest a dynamic phenotypic transition and/or the versatility of macrophages in endometriosis progression. Tet methylcytosine dioxygenase 3 (TET3) has recently emerged as a marker for pathogenic DAMs. TET3-overexpressing macrophages were found in endometriosis, and their depletion reduced endometriotic lesion growth in animal models. While these pathogenic DAMs differ in origins, surface/intracellular markers and other molecular features, TET3 overexpression underlies the definitive mechanism of the pathogenic properties in these DAMs. This study challenged the conventional emphasis on phenotypic markers like M1 and M2 markers and inspired further investigation into more underlying abnormalities in pathogenic DAMs [18].

A surprising similarity emerges when comparing macrophage phenotype transitions in endometriosis with those in tissue repair. After tissue injury, acute inflammation mediated by M1 macrophages is a necessary process, followed by M2 macrophage-mediated tissue repair. Both M1 and M2 macrophages are indispensable and their sequential transition guarantees a favorable microenvironment for successful tissue repair [19–21]. If the process of ectopic endometrium implantation and growth is regarded as a process of “endometrium repair at the wrong sites,” the disease pathogenesis may be explained from a tissue repair perspective. Pro-inflammatory macrophages in early-stage endometriosis resemble those seen in the initial phase of tissue injury, while the predominance of M2-like macrophages mirrors the pro-repair macrophages in the later stages of tissue repair. Macrophage-derived cytokines, such as VEGF and TGF-β1, also showed coincidence between endometriosis and tissue repair [22].

Endometriosis exhibits some characteristics of neoplasms, and they also share immunopathological similarities. Tumor-associated macrophages (TAMs) play a pivotal role in the tumor microenvironment (TME) and are divided by phenotype and function as pro-tumor (M2-like) and anti-tumor (M1-like) macrophages [23]. Research has shown that M2-like macrophages promote tumor cell proliferation, invasion, angiogenesis, and fibrosis, which ultimately enhance tumor growth. M2 TAMs exert immunomodulatory effects on other immune cells, such as cytotoxic T cells and NK cells, thereby dampening their cytotoxic effects and contributing to immune tolerance [24]. Ectopic endometrium also triggers a local immune response at the lesion site; therefore, escaping from the immune surveillance is necessary for lesion implantation and growth. The similarities between macrophages in endometriosis and TAMs imply that M2-like macrophage-mediated immune suppression and immune escape are common characteristics of both endometriosis and cancer.

Hormone imbalance in endometriosis contributes to macrophage dysfunction. Ectopic lesions overexpress estrogen and estrogen receptor β (ER-β). Hyperactivation of the ER-β signaling pathway results in MCP-1 secretion, recruiting macrophages to lesion sites. Estradiol (E2) also induces the production of inflammatory mediators including cytokines and prostaglandin E2 (PGE2). Progesterone normally restrains the effects of E2; however, ectopic lesions are mostly progesterone resistant as a result of reduced expression of progesterone receptor isoform B and isoform A (PR-B/PR-A) ratio, and progesterone resistance may further perturb the immune microenvironment [14].

### NK cells

NK cells are characterized by their cytotoxic functions that can rapidly target stressed or transformed cells and their cytokine-producing ability upon stimulation. Activation and inhibition of NK cells are intricately regulated by activating and inhibitory receptors and cytokines. The main characteristic of NK cells in endometriosis patients is reduced cytotoxicity [25]. NK cells are responsible for the recognition and frontline killing of ectopic endometrial cells. However, overexpression and over-activation of inhibitory receptors on NK cells limit their potential for cytotoxicity.

Many inhibitory receptors are increased in NK cells from endometriosis patients, including KIR2DL1 [26], CD94/NKG2A [27], and the leukocyte immunoglobulin-like receptors (LILRs) LILRB1/LILRB2 [28]. Overexpression of the inhibitory receptors on the peripheral blood NK (pNK) and peritoneal fluid NK (pfNK) cells indicates a reduced NK cell cytotoxicity at an overall systemic level and the peritoneal level. Notably, some ligands of the inhibitory receptors are highly expressed by endometriotic lesions. For example, human leukocyte antigen-E (HLA-E), the ligand of CD94/NKG2A and HLA-G, the ligament of LILRB1/LILRB2 are highly expressed by endometriotic lesions [27]. These self-recognitions render endometriotic lesions resistant to NK cell cytotoxicity through inhibitory receptor-mediated suppression, resulting in suppressed immunosurveillance and contributing to endometriotic cell implantation. In addition to direct cell-cell docking, ectopic stromal cells and macrophages also dampen cytotoxicity by paracrine secretion of IL-10 and TGF-β [29]. In contrast to inhibitory receptors with increased expression, the natural cytotoxicity receptor (NCR) NKp46 expression is significantly lower in pfNK in advanced endometriosis patients [30]. The reduced percentages of CD56 + NKp45 + and CD56dimNKp46 + NK cells were related to the reduced cytotoxicity of the pfNK cells in endometriosis, and the secretion of TNF-α and IFN-γ were increased. TNF-α and IFN-γ promote the proliferation of ectopic stromal cells and angiogenesis [31, 32]. Therefore, cytokine-producing NK cells further enhance endometriotic lesion implantation. A recent meta-analysis that included 31 studies provided a comprehensive perspective on NK cell alterations in endometriosis patients. No significant difference in the percentage of pNK cells or pfNK cells was detected between endometriosis patients and controls, while the percentage of uterine NK cells (uNK) was significantly increased in endometriosis patients. Meanwhile, this analysis confirmed that the cytotoxicity of pNK, pfNK, and uNK cells is significantly reduced in endometriosis patients [33].

Reduced NK cell activity was also demonstrated in animal models. In rats with induced endometriosis, NK cell activity in the spleen was significantly reduced compared with the sham group. Supernatants from endometriotic lesions and the peritoneal cavity also reduced NK cell activity [34]. Du et al. demonstrated that platelets protect endometriotic cells by forming a ‘pseudo-self’ coating on ectopic lesions and downregulating the activating receptor NKG2D ligands on these cells, thereby reducing NK cell cytotoxicity and protecting endometriotic lesions from elimination [35].

Single-cell RNA sequencing revealed that NK cells are enriched in endometriotic lesions compared with the eutopic endometrium [36]. NK cells from endometriotic lesions showed higher expression of *ENTPD1*, which encodes an immune tolerance receptor on uNK cells [6]. This evidence was consistent with previous clinical observations [28, 33]. The combined evidences strongly suggest that changes in NK cell frequency and cytotoxicity are key immunopathological features of endometriosis.

NK cells also exhibit similarities between endometriosis and cancer. A key feature of tumors is immune evasion, in which NK cell activity is suppressed by various molecules, cytokines, and inhibitory ligands in the TME. NK cell desensitization, whereby NK cells remain hyporesponsive despite the high expression of activating ligands on tumor cells, is another immune escape mechanism in tumors [37]. Although common in tumors, there is insufficient evidence to conclude whether desensitization also occurs in endometriosis.

### Dendritic cells (DCs)

DCs are specific antigen-presenting cells bridging the innate immune system and the adaptive immune system and playing important roles in both immune activation and tolerance. DCs are generally divided into conventional or classic DCs (cDCs), plasmacytoid DCs (pDCs), and monocyte-derived DCs (moDCs). DCs are classified as immature, semi-mature, or mature on the basis of phenotypical and functional properties. Immature DCs present “self” antigens to T cells in the absence of stimulatory signals, promoting immune tolerance, while mature DCs present “non-self” antigens with sufficient stimulatory signals to T and B cells, leading to immune clearance [38].

Schulke et al. showed that endometriosis patients had more immature DCs in the functional layer of the eutopic endometrium during both the proliferative and secretory phases compared with non-endometriotic women, while mature DC density was lower in both the basal and functional layers throughout the menstrual cycle. Additionally, immature DC density was higher in peritoneal endometriotic lesions compared with the eutopic endometrium of the same patients [39]. Seun et al. reported more pDCs highly expressing angiogenetic IL-10 in endometrioma lesions [40]. DCs were also detected in the peritoneal fluids from endometriosis patients. Izumi et al. reported that both cDCs and pDCs were detected in the peritoneal fluids from endometriosis and non-endometriosis patients, and mannose receptor (CD206)-positive conventional type 1 DCs were significantly increased in the endometriosis group [41]. This discovery was further verified by Guo and colleagues, who showed increased CD206 + DCs in the peritoneal fluids in stage I patients [10]. CD206 is an immature DC marker that facilitates the phagocytotic function of DCs [41]. Additionally, immunoglobulin receptor FceRIa expression was also elevated in early-stage endometriosis patients [10]. Given the role of FceRIa in activating Th2 cells and inducing antigen-specific T cell immune tolerance [10], these FceRIa-expressing DCs in the peritoneal fluid may promote lesion formation by facilitating immune tolerance to the ectopic cells. How the balance shifts towards immature DCs in the peritoneal fluid of endometriosis is unclear, but one explanation is that signals in the peritoneal fluid inhibit DC maturation [42]. These molecular signals may also cause systemic DC changes in patients. Mariada et al. observed that peripheral blood cDC1 cells showed a strong increasing trend in endometriosis patients, while the cDC2 population significantly declined [43]. Another study showed that the serum from endometriosis patients had higher levels of IL-10 and induced a tolerogenic DC phenotype in vitro [44]. Single-cell analysis revealed CD1C + DCs as the dominant DC population in eutopic endometrium and endometriosis lesions. Notably, one specific MSR1 + DC subset expressing immunomodulatory genes such as *MRC1* and *VSIG4* genes was identified in peritoneal lesions and the adjacent peritoneum, suggesting a possible role in immunotolerance [6].

The role of DCs in endometriosis has also been investigated in animal models, which consistently showed an abundance of DCs in induced lesions [44, 45]. Similar to results in human studies [39], Fainaru et al. found an enrichment of immature DCs in induced lesions, and peritoneal infusion of immature bone marrow-derived DCs enhanced angiogenesis and lesion growth [45]. However, ablation of CD11c + cDCs in transgenic mice by independent researches yielded conflicting results. Pencovich et al. observed reduced lesion growth [46], while Stanic et al. reported increased lesion sizes after DC ablation [47]. Considering the complexity of DC origins, phenotypes, and functions in diseases, more evidence from well controlled animal studies is needed to clarify the role of DCs in endometriosis.

### Granulocytes and mast cells

Neutrophils are frontline responders in immune reactions and crucial in pathogen defense and tissue injury. They also play a role in diseases like cancer, autoimmune disorders, and endometriosis. In the normal menstrual cycle, neutrophils increase significantly before menstruation, releasing matrix metalloproteinases that trigger endometrium rupture. Neutrophil-derived cytokines such as VEGF and IL-17 are indispensable for the subsequent endometrium repair. Circulating neutrophils from endometriosis patients displayed a more inflammatory transcriptomic profile compared with those from healthy women, indicating possible increased inflammatory responses under disease conditions. Increased neutrophils are also found in ectopic endometrium and peritoneal fluid, where they promote angiogenesis and lesion growth by releasing VEGF and other cytokines [40, 48]. Neutrophils release neutrophil extracellular traps (NETs) to fight against pathogens, but NETs also cause local inflammation and tissue damages. In endometriosis patients, circulating NETs and peritoneal fluid NETs were elevated, indicating a chronic inflammatory status [49]. Neutrophils in lesions also contribute to an immunosuppressive microenvironment by upregulating PD-L1 and FasL on themselves and/or other cells [50, 51]. In animal models, neutrophils were shown to be rapidly recruited to the peritoneum after lesion induction, contributing to increased chemokines and inflammatory and angiogenic cytokines. Blocking neutrophils early after induction reduced angiogenesis and lesion growth [48].

Eosinophils, basophils, and mast cells are also important in endometriosis. Eosinophils are rare in eutopic endometrium but accumulate before menstruation [52]. However, eosinophils are commonly detected in the ectopic endometrium at various phases and degranulating eosinophils are frequently detected especially at the fibrotic or perivascular areas; in normal endometrium, only few degranulating eosinophils are detected. Degranulating eosinophils have been related to high levels of inflammatory mediators such as eotaxin and IL-5, which boost eosinophil functions at lesion sites [53, 54].

Mast cells are stably detected in the endometrium during the menstrual cycle, but activation/degranulation occur before and during menstruation [52]. In endometriotic lesions, degranulated mast cells are more abundant during the proliferative and secretory phases, in contrast to the fewer, mostly granulated mast cells in eutopic endometrium of both endometriotic and non-endometriotic women [55]. In endometriosis, mast cells exhibit pro-inflammatory phenotypes in response to estrogen, and their interaction with endometriotic stromal cells may create a feedback loop that drives disease progression [56]. Studies on basophils in endometriosis are limited. However, a recent retrospective study by Feng et al. showed a positive correlation between higher basophil counts in peripheral blood and the risk of endometriosis, particularly in advanced stages (III/IV) [57].

## Adaptive immune cells in endometriosis

### T cells

T lymphocytes play a crucial role in the adaptive immune response and are categorized into CD4 + and CD8 + T cells. T cell dysregulation is a manifestation of endometriosis.

Depending on their function, CD4 + T cells can be classified into Th1, Th2, Th17, and Treg cells. On the basis of the produced cytokines and their functions, Th1 cells and Th17 cells are considered as pro-inflammatory and Th2 cells and Treg cells are considered anti-inflammatory.

The inflammatory cytokine interleukin-12 (IL-12) and TNF-α from other activated cells such as macrophages were shown to promote Th1 differentiation and conversely prevent Th2 differentiation. Subsequently, Th1 cells release IL-2, IL-12, and IFN-γ, etc. promoting cellular immunity and Th2 cell differentiation [58]. A study in an endometriosis mouse model showed that Th1 was increased in peritoneal fluid [59], and elevated IL-12 was associated with advanced endometriosis according to a cross-sectional survey [60]. Type 2 cytokines such as IL-4, IL-10 and IL-13 are useful in the production of Th2 cells. Endometriosis has been described as an immune shift towards Th2 as the increased type 2 cytokines detected in patients’ peritoneal fluids and serum [61, 62]. Intriguingly, the Th1/Th2 balance is similar to the M1/M2 balance. Pro-inflammatory cytokines may increase during the whole course of disease, while anti-inflammatory cytokines often appear in late stages of endometriosis and other chronic inflammatory diseases [63].

Abnormal Th17 behaviors also contribute to the inflammatory microenvironment. Research has shown that IL-17, the major pro-inflammatory cytokine produced by Th17 cells, was elevated in the plasma and peritoneal fluid of endometriosis patients, and IL-17 may be involved in endometriosis progression by stimulating angiogenesis and inflammatory cytokine release [64, 65]. Besides, abnormally elevated IL-17 and IL-23 levels in endometriosis play a role in female infertility [62, 66]. IL-10 + Th17 cells have also been observed in the endometriotic environment. These cells, triggered by IL-27 released from macrophages and endometrial stromal cells, progressively increase as endometriosis advances, further contributing to the inflammatory microenvironment [67].

An imbalance of Tregs (CD4 + CD25 + FOXP3+) contributes to the immune-suppressive microenvironment in endometriosis, promoting the establishment and maintenance of ectopic lesions. The results of studies on Tregs in endometriosis remain controversial, possibly because of heterogeneous methodology. Some clinical research found that FOXP3 + Treg cells were increased in lesions, decreased both in peripheral blood and peritoneal fluid, and increased in eutopic endometrium during the follicular phase in endometriosis patients. This was consistent with results in a baboon model, which showed an increase in Treg cells in ectopic lesions and decrease in peripheral blood and eutopic endometrium [68, 69]. In normal pregnancy, Tregs protect the fetus and its appendage tissues by inducing immune tolerance; abnormal eutopic Treg levels partly explain patient susceptibility to infertility and recurrent miscarriages [68]. In a case-control study including 55 endometriosis patients, increased FOXP3 + Treg in lesions showed a positive correlation with chronic pelvic pain [70]. These results indicate that Treg imbalance is related to local and systemic inflammation in endometriosis, and dysregulated Tregs contribute to the pathogenesis of endometriosis.

However, simply focusing on the FOXP3 + Treg cells is inadequate. Treg cells (CD4 + Foxp3 + T cells) have been divided into three subpopulations with heterogeneous functions: activated Tregs (aTreg, FOXP3hi CD45RA-); resting Treg (rTreg, FOXP3loCD45RA-) and non-suppressive Tregs (nTreg, FOXP3loCD45RA-) [71]. aTreg cells, the only subset with pure suppressive characteristics of Treg cells [72], inhibit the maturation of DCs by secreting the inhibitory cytokine IL-10, thus potentially promoting immunosurveillance escape and lesion establishment in peritoneal lesions [73]. rTreg differentiate into aTreg cells upon antigen/T cell receptor stimulation and exhibit strong immunosuppressive activity [74]. rTreg is also more active in TGFβ production compared with aTreg and thus may play a role in lesion fibrosis. nTreg, a mixture of follicular Tregs and cells producing pro-inflammatory cytokines, produce IL-17 to help B cell enrichment [73], thus promoting the formation of an immunosuppressive environment. Research on the three FoxP3 + cell fractions in endometriosis is limited. A comparative study revealed a significant reduction in the proportion of aTreg cells in ectopic lesions and eutopic endometrium compared with that observed in healthy women. Additionally, the progression of endometriosis was inhibited in Foxp3DTR/diphtheria toxin mice. Consistent with these findings, another study demonstrated that the proportion of aTregs was decreased in the eutopic endometrium during ovulation compared with healthy samples, but no significant difference was detected between the eutopic endometrium and peritoneal fluid in endometriosis group [75]. To better understand the role of Treg cells in endometriosis, clear definitions and specific characterizations of different subpopulations of Treg cells should be elucidated in future studies.

CD8 + T cells are enriched in endometriotic lesions compared with eutopic endometrium, with no differences in peripheral blood, and these cells do not vary with hormone levels [76]. CD8 + T cells, however, often showed a defective ability to clear ectopic endometriotic cells, and it was speculated that the CD8 T cells were predominantly exhausted CD8 + T cells [77], which is similar to T cell exhaustion in cancers [78]. CD8 + T cell functions are regulated by immune checkpoints (ICPs). A markedly elevated expression of the ICP PD-1/PD-L1 on T lymphocytes was detected in peripheral blood samples from endometriosis patients [79]. CLTA4, another ICP, was overexpressed in CD8 + T cells in the peripheral blood in endometriosis patients, and there was a positive correlation between the percentage of CLTA4 + T cells and disease severity [80]. This phenomenon represents a hallmark of the immune system’s reaction to chronic antigenic exposure in patients with endometriosis [81]. Oher classic ICPs such as TIM-3 and CD47 were also detected on T cells in endometriosis (see below “ICP based therapies”). Overexpression of ICPs restricts T cell cytotoxicity to endometriotic lesions, leads to T cell exhaustion, and finally contributes to immune evasion of ectopic lesions.

### B cells

There are limited studies and need further exploration regarding the role of B cells in endometriosis. Several reports have shown that patients with endometriosis have increased and activated B lymphocytes (CD23+) and excessive production of autoantibodies [82], mimicking autoimmune diseases. The B-cell lymphoma 6 (*BCL6*) gene is essential for B cell maturation and is overexpressed in endometriotic lesions [83], suggesting overactive B cell maturation at the lesion sites. Single-cell analysis of menstrual endometrial tissues also revealed a notable rise in B cells within the shed endometrium of endometriosis patients [84]. Elevated levels of IgG and IgA were observed in the peritoneal fluid of women with endometriosis [30], and they trigger the peritoneal immune responses. Approximately 60% of endometriosis patients experiencing infertility test positive for autoantibodies, with p53 as the most frequent [85]. However, the extent to which B cells and autoantibodies play a role in the development and progression of endometriosis remains inconclusive. A common opinion is that the abnormal levels and activation of B lymphocytes result in immune evasion of endometrial cells, thereby hastening the advancement of the disease [61]. Additionally, B cells can suppress inflammatory immune responses and promote endometrial cell proliferation in endometriosis by secreting IL-10 and IL-35 [86]. Studies in a mouse model showed that B lymphocyte inactivation limited endometriosis development and fibrosis [87, 88].

The involvement of immune system in the pathophysiology of endometriosis is extensive. Immune cells exhibited altered behaviors in the milieu of endometriosis; more importantly, immune cells also exert influences on each other by secreting cytokines and/or direct cell-cell docking (Fig. 2). Elucidating the complicated interaction network of immune cells would lead to better understanding of the immunopathology of endometriosis.

## Endometriosis-associated inflammation influences female fertility: from the ovary to delivery

Endometriosis significantly impairs fertility. Aberrant inflammatory cytokines and immune cell dysfunction drive systemic inflammation, which is increasingly recognized as a major contributor to infertility in endometriosis patients (Fig. 3).

### Impaired ovarian function, follicle development and oocyte quality

Endometrioma, or ovarian endometriosis, can directly and indirectly damage ovarian tissue and function. Overgrowing endometriomas physically compromise the surrounding ovarian tissue, and the chaotic components of endometrioma induce considerable local and systemic inflammation [89]. Studies have reported aberrant concentrations of cytokines such as VEGF, IL-8, IL-6, and TNF-α in endometrioma cyst fluid compared with other benign cysts [90]. The unabsorbed blood within endometrioma results in high iron concentrations, which disrupt redox homeostasis and catalyze the propagation of reactive oxygen species (ROS), exacerbating local inflammation [91]. Endometriomas may affect follicle development through an inflammatory mechanism. Follicular fluids from endometriosis patients contain higher levels of inflammatory cytokines (IL-1β, IL-6, IL-8, TNF-α, MCP-1), suggesting that endometriomas cause inflammatory changes to surrounding follicles [91, 92]. ROS levels are also elevated in follicle fluids [91]. Dysregulated inflammatory cytokines such as TNF-α and excessive ROS both directly interfere with follicle development and, more severely, cause oocyte damage [90, 93], which may account for compromised follicular responses and lower oocyte yields during IVF in these patients. Other types of endometriosis also show systemic, peritoneal, and pelvic inflammatory changes and redox imbalance, which may also impact follicle and oocyte quality in a similar pattern [94].

### Unhospitable pelvic microenvironment for fertilization and reduced embryo quality

Endometriosis is positively associated with chronic pelvic inflammatory disease (PID) [95]. PID patients with endometriosis manifest more severe symptoms and often require surgical interventions. Chronic inflammation in the pelvic cavity may impair reproductive organs and create an inhospitable environment for sperm, reducing their motility and fertilization ability [96]. Animal experiments further indicated that excessive inflammatory cytokines and high ROS levels hindered zygote migration and development [97]. Murine zygotes exposed to peritoneal fluids from endometriosis women exhibited poor proliferation and increased apoptosis [98, 99]. Collectively, these findings indicate that endometriosis-associated inflammation disrupts ovary function, fertilization, and embryo development.

### Reduced endometrium receptivity and unfavorable maternal-fetal interface

Apart from ovary damage and incompetent blastocysts, endometriosis infertility was also related with insufficient endometrium receptivity and an unfavorable maternal-fetal interface. While the mechanism underlying implantation failure in endometriosis is still under debate, hormonal reactivity disruption is commonly discussed. Similar to the estrogen dominance and progesterone resistance in ectopic lesions [2], gene-expression profiling for endometriosis patients revealed a decreased number of progesterone-target genes involved in decidualization during the window of embryo implantation in eutopic endometrium [100]. Epigenetic changes in eutopic endometrium such as homeobox protein Hox-A10 hypermethylation inhibit the decidualization process [101]. Decidual NK cells (dNKs) are the main immune cells regulating embryo implantation in the eutopic endometrium. However, dNK cells in endometriosis patients exhibited compromised maturation and increased cytotoxicity during the window of implantation, resulting in damaged endometrium receptivity and subsequent implantation failure [102]. These abnormal behaviors of NK cells may also be attributed to dysregulated estrogen. In studies in mouse models, Lai et al. showed that estrogen antagonists improved dNK cell functions and improved endometriosis-associated implantation failure [103]. Increased M1 macrophages in the secretory phase in eutopic endometrium in endometriosis disrupts the microenvironment for implantation, which indicates recurrent implantation failure [104, 105]. These factors collectively contribute to the infertility and miscarriage observed in patients with endometriosis. Disturbances of the adaptive immune system also participate in reduced endometrial fertility. Activated B lymphocytes in endometriosis produce excessive antibodies against endometrial antigens, anti-laminin-1 auto-antibodies, and other auto-immune antibodies (e.g., antiphospholipid), which may attack the maternal-fetal interface and result in recurrent miscarriages [106].

### Increased risks for obstetric complications and poor pregnancy outcomes

Endometriosis is related to relatively higher risks of pregnancy complications and poor outcomes. Clinical studies have reported the associations of endometriosis with miscarriage, pre-eclampsia, placenta previa, preterm pre-labor rupture of membranes, preterm birth, and cesarean delivery, especially in women with a history of deep infiltrating endometriosis [107, 108]. Evidence from animal and clinical studies demonstrated that chronic inflammation is related to many of these events [109]. For example, high levels of inflammatory mediators such as cytokines and prostaglandins can induce preterm labor contractions, and inflammatory macrophages in eutopic endometrium may contribute to miscarriage [104]. While the mechanisms underlying these associations are complex, the inflammatory process is one possible link between endometriosis and adverse pregnancy outcomes. The correlation between adverse pregnancy outcomes and endometriosis has been mostly demonstrated in retrospective studies. Clinical indications regarding pregnancy in endometriosis patients should be considered with caution due to the substantial heterogeneity of the studies [107, 110]. Hence, well-controlled large-scale prospective cohort studies are necessary to provide higher levels of evidence to guide obstetrical practices.

## Immunological therapeutic targets of endometriosis

The current treatments for endometriosis include nonsteroidal anti-inflammatory drugs(NSAIDs), progestin-based therapy, GnRH agonists, aromatase inhibitors, and surgery. These classic treatments are effective in most patients [1]. Better understanding of the immune and inflammatory aspects of endometriosis may not only help reveal the immunological effects of traditional treatments but also lead to the development of novel therapeutic strategies.

### Immune cell therapies

As discussed above, immune cell dysfunction contributes to endometriosis development. Elimination or transfusion of certain immune cells, such as macrophages and NK cells, significantly influenced endometriosis progression in animal models [16, 28], indicating the promise of immune cell therapy in endometriosis. Pathogenic DAMs associated with endometriosis progression express multiple markers including CD163 and CD206, which may be targets for targeted therapies. Reprogramming macrophages from pro-endometriosis to anti-endometriosis activity has also proven effective in animals [111]. Restoration of NK cell activity is another potential option. Blocking NK cell inhibitory receptors may overcome NK cell inhibition and boost their cytotoxicity [28]. Transfusion of ex vivo stimulated autologous anti-disease macrophages or NK cells may also be promising therapies. As for T cell dysfunctions, available immune checkpoint inhibitors (ICIs, see below) may be safe and powerful agents for endometriosis, which is supported by increasing evidence.

### Immune checkpoint (ICP)-based therapies

Originally developed for cancer treatment, ICP-based therapies have drawn attention as endometriosis treatments because of the similarities between the immune microenvironments of cancer and endometriosis. Dysregulation of key ICP pathways, such as PD-1/PD-L1, TIM-3/Gal-9, CTLA-4/CD80/CD86, and CD47/SIRPα, has been documented in endometriosis [112]. Aberrant ICP expression has been detected in the ectopic endometrium, immune cells, serum and peritoneal fluids in endometriosis patients [112]. Endometriotic stromal cells proactively modulate immune cells to favor their growth by expressing ICP ligands or receptors including PD-1, PD-L1/2, FasL, and CD47 or by inducing their expressions on other cells [113, 114]. Disturbances in ICP signaling in endometriosis contribute to immune evasion similar to cancer, suggesting that ICIs, effective in oncology, may hold potential for endometriosis treatment. Promising studies showed that anti-PD-L1 and anti-CTLA4 treatment reduce endometriotic lesion size and inflammation levels in animal models [114, 115]. More research with animal experiments and clinical trials should be pursued to support the repurposing of ICIs for endometriosis.

### Cytokine-targeting therapies

Aberrant cytokines, a prominent feature of endometriosis, are important mediators of the immune microenvironment. Inflammatory cytokines such as TNF-α, IL-1, and IL-6 have various effects on immune cells and other cells, playing important roles in sustaining inflammation. Other cytokines such as VEGF and TGF-β1 promote lesion angiogenesis and fibrosis. VEGF activates vascular endothelial cells and enhances lesion vascularization [116]; TGF-β1 and other pro-fibrotic factors together facilitate the myofibroblast transformation of endometriotic stromal cells [117, 118]. These cytokines, as the main hub of immunopathogenic processes, are potentially potent therapeutic targets. Blockade of these cytokines using antibodies or inhibitors has been shown to reduce inflammation, angiogenesis, and fibrosis in endometriosis and ultimately ameliorate disease [116, 119].

## Other therapies with immune-modulating effects for endometriosis

### Hormone therapies

Progestin-based therapy is the first-line therapy for endometriosis. Progestins counteract estradiol and directly induce apoptosis of endometriotic cells and lesion atrophy. Progestins also regulate the immune microenvironment. Macrophages express progesterone receptors, and progestins suppress macrophage activation and inhibit the release of inflammatory cytokines such as IL-1β, IL-6, MCP-1 and TNF-α [120]. While the secretion of these cytokines is usually associated with classically activated macrophages, other studies show that progestins suppress not only classic activation but also alternative activation of macrophages [121], which further suggests that alternatively activated macrophages are a potent therapeutic target for endometriosis. Progestins may also enhance NK cell activity. GnRH agonists and aromatase inhibitors suppress endogenous estradiol concentrations, thus inhibiting lesion growth and lowering estrogen-dependent inflammation in endometriosis.

### Immune-modulating small molecules

COX1/2 inhibitors, one type of NSAIDs, are traditionally used to manage endometriosis associated pain by reducing local prostaglandins. Inhibition of COX1/2 also inhibits VEGF signaling, thus inhibiting angiogenesis in endometriotic lesions [122]. Recent findings showed that COX1/2 inhibitors disrupted macrophage-mediated T cell suppression, enhancing ICI efficiency in tumors, which might inspire new studies of small molecule COX1/2 inhibitors in endometriosis [123]. Similarly, targeting TET3 overexpressing macrophages with small molecules like Bobcat339, which promotes TET3 degradation, represents another potential therapeutic reagent [18].

### Dietary interventions and alternative therapies

Nutrients such as vitamin B, polyphenols, and antioxidants like vitamins C and E have been shown to reduce systemic inflammation in endometriosis patients [124]. Traditional Chinese acupuncture triggers the anti-inflammatory reflex, and clinical studies proved that acupuncture helped relieve pelvic pain and improved other symptoms in endometriosis patients [125]. These studies indicated that alternative therapies influence inflammation status and may be considered in endometriosis management.

### Summary and remarks

With improved understanding of the pathophysiology, endometriosis should be recognized as a systemic disease rather than a pelvic-restricted disease. Local and systemic immunopathological changes are prominent features, as well as possible disease initiators and/or sustainers. Immune cell dysfunctions, aberrant inflammatory mediators, and disrupted ICP signaling together promote an inflammatory and immune-suppressive microenvironment for endometriosis development (Figs. 1 and 2). Endometriosis-associated inflammation significantly sabotages female fertility and pregnancy on a scale from ovary to delivery (Fig. 3). Research on the immunopathological changes in endometriosis prompt the discovery of new therapeutic targets. Immunopathological similarities between endometriosis and cancers are intriguing, as they provide novel perspectives for understanding and treating endometriosis. Translating the research achievements of ICPs and ICIs from cancer to endometriosis is a remarkable advance, and more evidence should be expected to support the repurposing of ICIs. Other promising immunological treatments for endometriosis include immune-regulating small molecules, immune cell therapies, cytokine-targeting therapies, and alternative therapies. However, these novel therapies should be supported by future large-scale, well-controlled, stage-stratified clinical trials, and the question should be addressed whether the benefits of new therapies outweigh the expenses and risks of adverse effects compared with current treatments. Reduced fertility is another main concern of endometriosis. Most research has focused on immunological changes and the effects of immunological interventions on endometriotic lesions. Additional efforts should address whether targeting inflammation will also be beneficial for fertility in endometriosis patients.

## Data Availability

No additional data generated in this work.
